# Supplementary material for: Real-time impacts of air pollution on the health, well-being, and daily life of children and young people in Delhi and Dhaka
Source: PLOS Glob Public Health. 2026 Jun 23;6(6):e0005382. doi: 10.1371/journal.pgph.0005382 (PMC13289869; doi:10.1371/journal.pgph.0005382)
Supplement: S1 File — Examples of the digital advertisements used to recruit participants in Delhi and Dhaka via Meta and Google platforms. (DOCX) [file pgph.0005382.s002.docx]

**S1 File: Survey Recruitment and Digital Advertisement Strategies in Delhi and Dhaka**

**Table 1:** Platforms and Formats for Digital Recruitment

| Advertising Platforms | Advertising Networks | Advertisement Formats |
| --- | --- | --- |
| Google | YouTube, Gmail, Google Search, Google Display, Google Discover | Text, Images, Video |
| Meta | FaceBook, Instagram | Image, Video, Stories, Carousel, Reels |

**Table 2:** Cities and Languages of Advertisement Campaigns

| **City** | **Language of Advertisement** |
| --- | --- |
| Dhaka | Bengali (Bangla) |
| Delhi | Hindi |

.
