# Supplementary material for: Real-time impacts of air pollution on the health, well-being, and daily life of children and young people in Delhi and Dhaka
Source: PLOS Glob Public Health. 2026 Jun 23;6(6):e0005382. doi: 10.1371/journal.pgph.0005382 (PMC13289869; doi:10.1371/journal.pgph.0005382)
Supplement: S2 Text — Full survey questionnaire in Hindi, as used in Delhi. (DOCX) [file pgph.0005382.s003.docx]

# S 2 Text: Consent text and Data collection tool in Hindi

# हिंदी अनुवाद

ड्राफ्ट भर्ती विज्ञापन:
(बच्चों, शहरों और जलवायु से संबंधित फेसबुक, इंस्टाग्राम और गूगल अकाउंट के माध्यम से 'प्रमोटेड कंटेंट' के रूप में साझा किया जाएगा)

नमस्ते, हम LSHTM से शोधकर्ता हैं और हम यह समझना चाहते हैं कि आज आप कैसा महसूस कर रहे हैं और क्या कर रहे हैं। यह 2-मिनट का सर्वेक्षण आपके शहर के 29 वर्ष से कम उम्र के युवाओं और 18 वर्ष से कम उम्र के बच्चों के माता-पिता के लिए है, जो उच्च वायु प्रदूषण की अवधि के दौरान या उसके तुरंत बाद है। सभी डेटा गुमनाम होंगे। हम गुमनाम परिणामों का विश्लेषण करेंगे और उन्हें जनता, अन्य शोधकर्ताओं और नीति निर्माताओं के साथ साझा करेंगे। आप किसी भी समय रुक सकते हैं और lshtm.ac.uk/ccc/info पर अधिक जानकारी प्राप्त कर सकते हैं। क्या आप इस सर्वेक्षण में भाग लेना चाहेंगे?*
हाँ
नहीं

स्क्रीन 2
आपकी आयु कितनी है? (वर्षों में)*

स्क्रीन 3
क्या आप 18 वर्ष से कम आयु के बच्चे के माता-पिता हैं?*
हाँ
नहीं

स्क्रीन 4
यदि आप माता-पिता हैं, तो कृपया इन प्रश्नों का उत्तर देते समय अपने सबसे छोटे बच्चे को ध्यान में रखें।
यदि आप माता-पिता नहीं हैं, तो केवल अपने लिए उत्तर दें।

स्क्रीन 5
आपके सबसे छोटे बच्चे की आयु कितनी है?*
(जिस बच्चे के लिए आप यह सर्वेक्षण पूरा कर रहे हैं उसकी आयु वर्षों में लिखें। यदि आपका बच्चा 1 वर्ष से कम है, तो "0" टाइप करें)

स्क्रीन 6
यदि आप माता-पिता हैं, तो कृपया अपने सबसे छोटे बच्चे को ध्यान में रखें।
यदि आप माता-पिता नहीं हैं, तो केवल अपने लिए उत्तर दें।

स्क्रीन 7
आज आप (या यदि आप माता-पिता हैं तो आपका सबसे छोटा बच्चा) कैसा महसूस कर रहा है?
 बहुत अच्छा
अच्छा
ठीक
बुरा
बहुत बुरा

स्क्रीन 8
पिछली रात, कुल मिलाकर, आपने (या यदि आप माता-पिता हैं तो आपके सबसे छोटे बच्चे ने) कितनी अच्छी नींद ली?
 बहुत अच्छा
अच्छा
ठीक
बुरा
बहुत बुरा

स्क्रीन 9
पिछले 24 घंटों में, क्या आपने (या यदि आप माता-पिता हैं तो आपके सबसे छोटे बच्चे ने) निम्नलिखित लक्षणों में से कोई अनुभव किया है? (अगले प्रश्न पर जाने के लिए सभी का उत्तर दें)

|  | हाँ | नहीं |
| --- | --- | --- |
| आँखों में खुजली |  |  |
| गले में खराश |  |  |
| खांसी |  |  |
| त्वचा में जलन/दाने |  |  |
| दस्त या उल्टी |  |  |
| श्वसन कठिनाई |  |  |
| कम मूड |  |  |
| चिंता / तनाव |  |  |
| काम या स्कूल में ध्यान केंद्रित करने में कठिनाई |  |  |
| सिरदर्द |  |  |
|  |  |  |

स्क्रीन 10
पिछले 24 घंटों में आपने (या आपके सबसे छोटे बच्चे ने) लगभग कितने मिनट शारीरिक गतिविधि की?
- 0 मिनट
- 1-15 मिनट
- 16-30 मिनट
- 31-45 मिनट
- 46-60 मिनट
- 60 मिनट से अधिक

स्क्रीन 11
क्या उच्च वायु प्रदूषण के कारण पिछले 24 घंटों में आपके (या आपके सबसे छोटे बच्चे के) साथ इनमें से कोई घटना हुई?

|  | हाँ | नहीं |
| --- | --- | --- |
| स्कूल या काम के लिए देर हुई |  |  |
| स्कूल या काम पूरी तरह छूट गया |  |  |
| महत्वपूर्ण बैठक या साक्षात्कार छूट गया |  |  |
| स्वास्थ्य देखभाल अपॉइंटमेंट छूट गया |  |  |
| दोस्तों / परिवार से मिलने का प्लान रद्द किया |  |  |
| घर में पर्याप्त भोजन नहीं था |  |  |
| स्वच्छ पेयजल तक पहुँच नहीं हुई |  |  |
| परिवार की अधिक सहायता की आवश्यकता (जैसे बुजुर्ग रिश्तेदारों की मदद करना, स्कूल नहीं जाने वाले बच्चों की देखभाल करना) |  |  |

स्क्रीन 12
उच्च वायु प्रदूषण के बारे में आप कितने चिंतित हैं?
१ २ ३ ४ ५ ६ ७ ८ ९ १०

बिल्कुल भी चिंतित नहीं मध्यम चिंतित अत्यधिक चिंतित

स्क्रीन 13
उच्च वायु प्रदूषण के लिए आपके समुदाय की तैयारी और प्रतिक्रिया से आप कितने संतुष्ट हैं?
 बहुत संतुष्ट
 कुछ हद तक संतुष्ट
 तटस्थ
 कुछ हद तक असंतुष्ट
 बहुत असंतुष्ट

स्क्रीन 14
यदि आप अपने शहर/कस्बे को स्वस्थ और अधिक टिकाऊ बनाने के लिए एक काम कर सकते हैं, तो वह क्या होगा? (वैकल्पिक)

स्क्रीन 15
आपका (या आपके सबसे छोटे बच्चे का) लिंग क्या है?
- महिला
- पुरुष
- अन्य / बताना नहीं चाहते

स्क्रीन 16
आपके परिवार की कुल मासिक आय लगभग कितनी है? (अमेरिकी डॉलर में)
- <$100
- $100 से $499
- $500 से $1499
- $1500 से $4000
- >$4000

स्क्रीन 17
कुछ दिन पहले, हमने एक समान सर्वेक्षण किया था। क्या आपने पहले इस अध्ययन में भाग लिया था?
हाँ
नहीं
धन्यवाद आपके समय के लिए! यदि आपके कोई प्रश्न हैं, तो कृपया ccc@lshtm.ac.uk पर ईमेल करें।
