# Supplementary material for: Real-time impacts of air pollution on the health, well-being, and daily life of children and young people in Delhi and Dhaka
Source: PLOS Glob Public Health. 2026 Jun 23;6(6):e0005382. doi: 10.1371/journal.pgph.0005382 (PMC13289869; doi:10.1371/journal.pgph.0005382)
Supplement: S3 Text — Full survey questionnaire in Bengali, as used in Dhaka. (DOCX) [file pgph.0005382.s004.docx]

## S 3 Text: Consent text and Data collection tool in Bengali

# বাংলা অনুবাদ

নিয়োগ বিজ্ঞাপন খসড়া:
(এই বিজ্ঞাপনটি 'প্রমোটেড কন্টেন্ট' হিসেবে শিশু, শহর এবং জলবায়ু সম্পর্কিত ফেসবুক, ইনস্টাগ্রাম এবং গুগল অ্যাকাউন্টে শেয়ার করা হবে)

হ্যালো, আমরা LSHTM-এর গবেষক এবং আমরা জানতে চাই আজ আপনি কেমন অনুভব করছেন এবং কী করছেন। এই ২-মিনিটের জরিপটি আপনার শহরের ২৯ বছরের কম বয়সী যুবক-যুবতীদের জন্য এবং ১৮ বছরের কম বয়সী শিশুদের বাবা-মায়েদের জন্য, যখন বায়ু দূষণের মাত্রা খুব বেশি থাকে বা তার ঠিক পরে। সমস্ত তথ্য গোপনীয় রাখা হবে। আমরা গোপনীয় ফলাফল বিশ্লেষণ করব এবং সেগুলি জনগণ, অন্যান্য গবেষক এবং নীতি নির্ধারকদের সাথে ভাগ করব। আপনি যেকোনো সময় অংশগ্রহণ বন্ধ করতে পারেন এবং lshtm.ac.uk/ccc/info থেকে আরও তথ্য পেতে পারেন। আপনি কি এই জরিপে অংশগ্রহণ করতে চান?*
হ্যাঁ
না

স্ক্রিন ২
আপনার বয়স কত? (বছরে উল্লেখ করুন)*

স্ক্রিন ৩
আপনি কি ১৮ বছরের কম বয়সী শিশুর বাবা-মা?*
হ্যাঁ
না

স্ক্রিন ৪
যদি আপনি বাবা-মা হন, অনুগ্রহ করে এই প্রশ্নগুলোর উত্তর দেওয়ার সময় আপনার কনিষ্ঠ শিশুকে বিবেচনা করুন।
যদি আপনি বাবা-মা না হন, কেবল নিজের জন্য উত্তর দিন।

স্ক্রিন ৫
আপনার কনিষ্ঠ সন্তানের বয়স কত?*
(যে শিশুর জন্য আপনি এই জরিপটি পূরণ করছেন তার বয়স বছর হিসাবে উল্লেখ করুন। আপনার শিশু যদি ১ বছরের কম হয় তবে "০" টাইপ করুন)

স্ক্রিন ৬
যদি আপনি বাবা-মা হন, অনুগ্রহ করে আপনার কনিষ্ঠ শিশুকে বিবেচনা করুন।
যদি আপনি বাবা-মা না হন, কেবল নিজের জন্য উত্তর দিন।

স্ক্রিন ৭
আজ আপনি (বা আপনার কনিষ্ঠ শিশু যদি আপনি বাবা-মা হন) কেমন বোধ করছেন?
খুব ভালো
 ভালো
 ঠিক আছে
 খারাপ
 খুব খারাপ

স্ক্রিন ৮
গত রাতে আপনি (বা আপনার কনিষ্ঠ শিশু যদি আপনি বাবা-মা হন) কতটা ভালো ঘুমিয়েছেন?
 খুব ভালো
 ভালো
 ঠিক আছে
 খারাপ
 খুব খারাপ

স্ক্রিন ৯
গত ২৪ ঘন্টায় আপনি (বা আপনার কনিষ্ঠ শিশু যদি আপনি বাবা-মা হন) নিম্নলিখিত কোনো উপসর্গ অনুভব করেছেন কি? (পরবর্তী প্রশ্নে যেতে সবগুলোর উত্তর দিন)

|  | হ্যাঁ | না |
| --- | --- | --- |
| চুলকানি চোখ - গলা ব্যথা - কাশি - ত্বকের জ্বালা বা র‍্যাশ - ডায়রিয়া বা বমি - শ্বাসকষ্ট - মন্দ মেজাজ - উদ্বেগ / চাপ - কাজ বা স্কুলে মনোযোগ কেন্দ্রীভূত করতে অসুবিধা - মাথাব্যথা |  |  |

স্ক্রিন ১০
গত ২৪ ঘন্টায় আপনি (বা আপনার কনিষ্ঠ শিশু) কত মিনিট শারীরিক কার্যকলাপ করেছেন?
- ০ মিনিট
- ১-১৫ মিনিট
- ১৬-৩০ মিনিট
- ৩১-৪৫ মিনিট
- ৪৬-৬০ মিনিট
- ৬০ মিনিটের বেশি

স্ক্রিন ১১
উচ্চ বায়ুদূষণের কারণে গত ২৪ ঘন্টায় আপনার (বা আপনার কনিষ্ঠ শিশুর) সাথে নিম্নলিখিত ঘটনা ঘটেছে কি? (পরবর্তী প্রশ্নে যেতে সবগুলোর উত্তর দিন)

|  | হ্যাঁ | না |
| --- | --- | --- |
| স্কুল বা কাজে দেরি হয়েছে |  |  |
| স্কুল বা কাজ পুরোপুরি মিস হয়েছে |  |  |
| গুরুত্বপূর্ণ মিটিং বা সাক্ষাৎকার মিস হয়েছে |  |  |
| স্বাস্থ্য সেবা অ্যাপয়েন্টমেন্ট মিস হয়েছে |  |  |
| বন্ধু বা পরিবারের সাথে দেখা করার পরিকল্পনা বাতিল হয়েছে |  |  |
| ঘরে পর্যাপ্ত খাদ্য ছিল না |  |  |
| বিশুদ্ধ পানীয় জল পাওয়া যায়নি |  |  |
| পরিবারের বাড়তি সহায়তার প্রয়োজন |  |  |

স্ক্রিন ১২
উচ্চ বায়ুদূষণ নিয়ে আপনি কতটা উদ্বিগ্ন?
১ ২ ৩ ৪ ৫ ৬ ৭ ৮ ৯ ১০

কোনো উদ্বেগ নেই মাঝারি উদ্বেগ অত্যন্ত উদ্বিগ্ন

স্ক্রিন ১৩
উচ্চ বায়ুদূষণের জন্য আপনার সম্প্রদায়ের প্রস্তুতি এবং প্রতিক্রিয়ার সাথে আপনি কতটা সন্তুষ্ট?
 খুব সন্তুষ্ট
 কিছুটা সন্তুষ্ট
 নিরপেক্ষ
 কিছুটা অসন্তুষ্ট
 খুব অসন্তুষ্ট

স্ক্রিন ১৪
আপনি যদি আপনার শহর/শহরকে আরও স্বাস্থ্যকর এবং টেকসই করতে একটি কাজ করতে পারতেন, তবে তা কী হতো? (ঐচ্ছিক)

স্ক্রিন ১৫
আপনার (বা আপনার কনিষ্ঠ শিশুর) লিঙ্গ কি?
- মহিলা
- পুরুষ
- অন্যান্য / বলতে অনিচ্ছুক

স্ক্রিন ১৬
আপনার পরিবারের মোট মাসিক আয় কত? (মার্কিন ডলার)
- <$100
- $100 থেকে $499
- $500 থেকে $1499
- $1500 থেকে $4000
- >$4000

স্ক্রিন ১৭
কয়েক দিন আগে আমরা অনুরূপ একটি জরিপ করেছি। আপনি কি আগে এই জরিপে অংশগ্রহণ করেছেন?
হ্যাঁ
না
ধন্যবাদ আপনার সময়ের জন্য! কোনো প্রশ্ন থাকলে দয়া করে ccc@lshtm.ac.uk-এ ইমেল করুন।
