# Supplementary figures and images for: Real-time impacts of air pollution on the health, well-being, and daily life of children and young people in Delhi and Dhaka
Source: PLOS Glob Public Health. 2026 Jun 23;6(6):e0005382. doi: 10.1371/journal.pgph.0005382 (PMC13289869; doi:10.1371/journal.pgph.0005382)

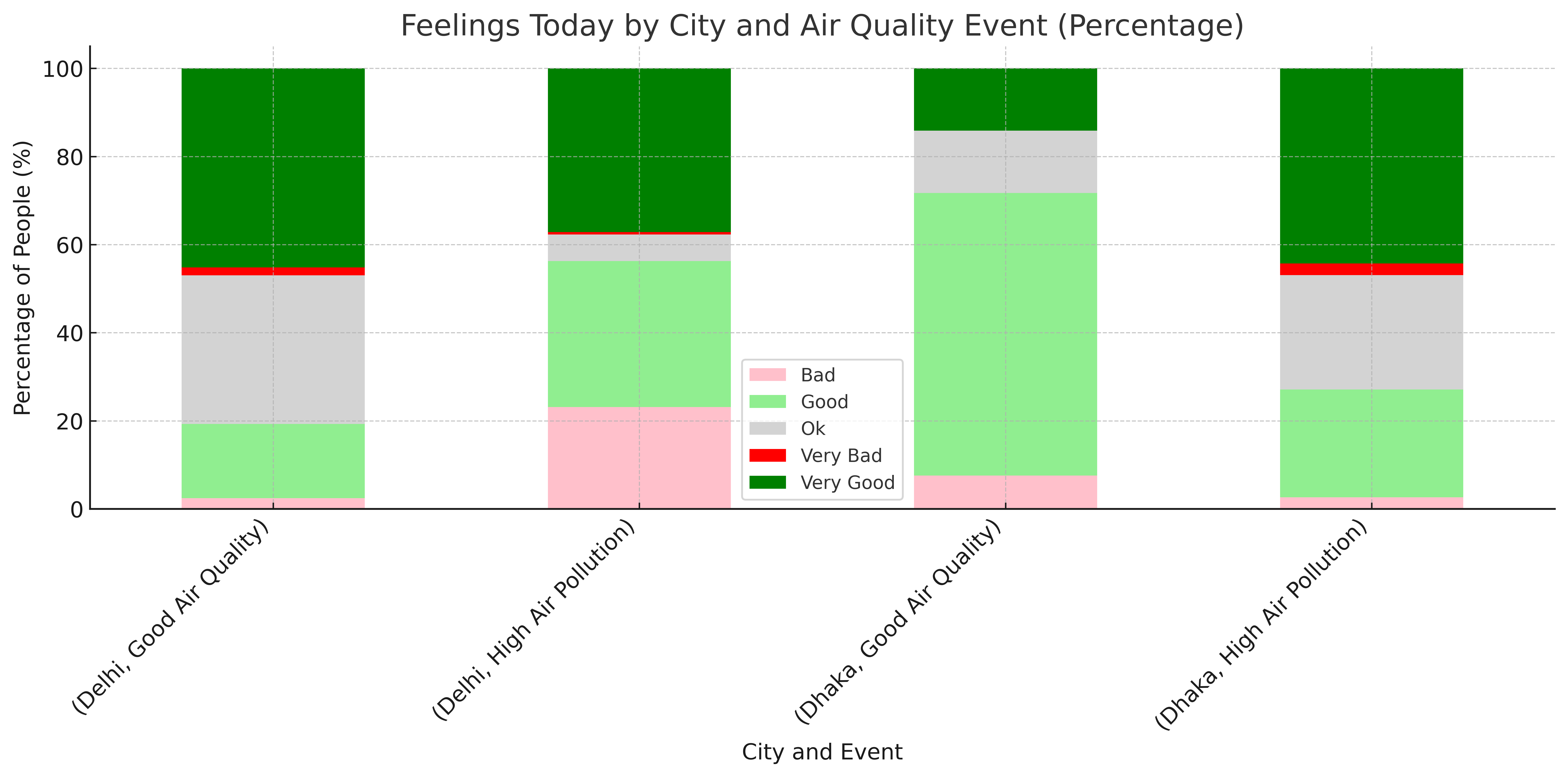

Supplement: S1 Fig — Figure showing the percentage of participants reporting general feelings during good air quality and high air pollution periods per city. (TIF) [file pgph.0005382.s007.tif]

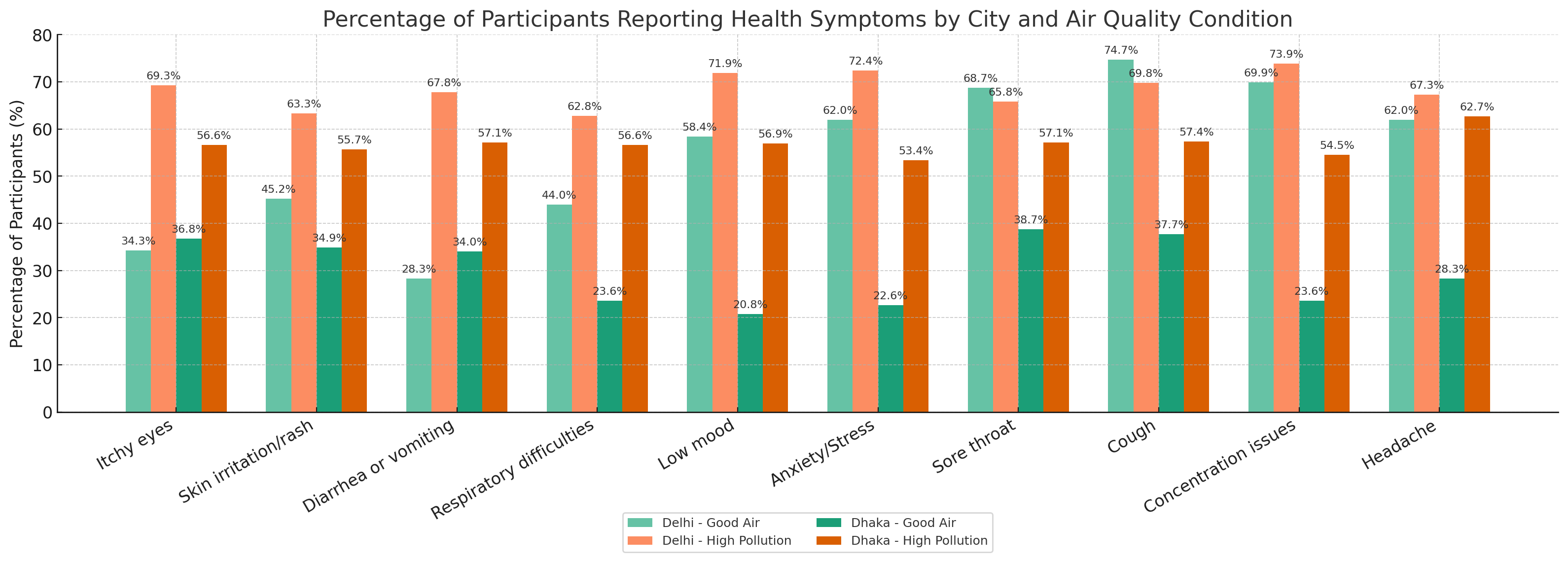

Supplement: S2 Fig — Figure showing the percentage of participants reporting physical and emotional health symptoms during good and high air pollution periods per city. (TIF) [file pgph.0005382.s009.tif]

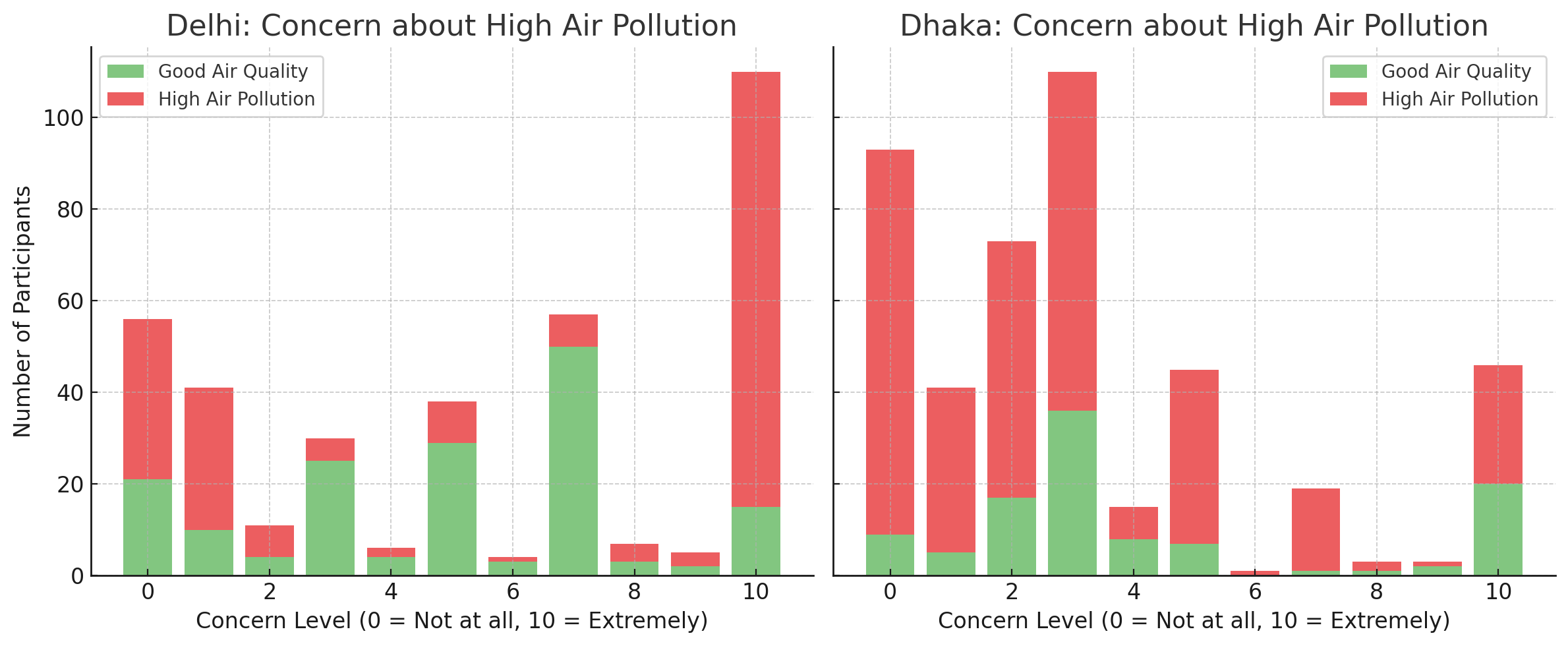

Supplement: S3 Fig — Figure showing the distribution of participant concern levels in Delhi and Dhaka across air quality periods. (TIF) [file pgph.0005382.s016.tif]

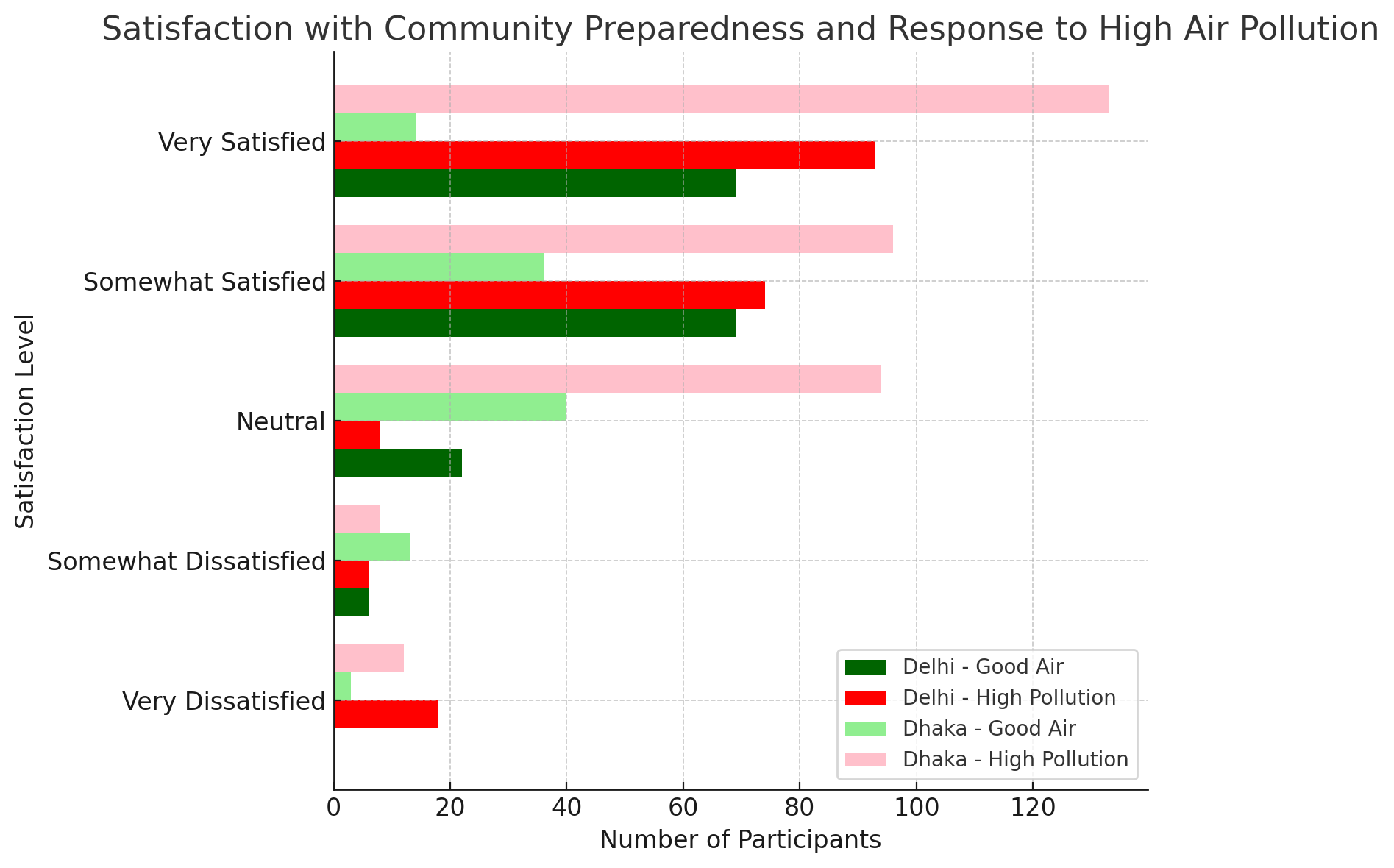

Supplement: S4 Fig — Figure showing participant satisfaction with city preparedness and response to high air pollution in Delhi and Dhaka. (TIF) [file pgph.0005382.s018.tif]
