# Supplementary material for: Real-time impacts of air pollution on the health, well-being, and daily life of children and young people in Delhi and Dhaka
Source: PLOS Glob Public Health. 2026 Jun 23;6(6):e0005382. doi: 10.1371/journal.pgph.0005382 (PMC13289869; doi:10.1371/journal.pgph.0005382)
Supplement: S4 Table — Adjusted odds ratios for the likelihood of experiencing symptoms during high-pollution compared with good air quality periods. (DOCX) [file pgph.0005382.s011.docx]

**S4 Table: Binary logistic regression examining the relationship between air quality conditions, demographic characteristics, and the presence of specific health symptoms.**

This table presents adjusted odds ratios from binary logistic regression models assessing associations between air quality events (Good Air Quality vs. High Air Pollution), demographic characteristics, and the presence of selected health symptoms. Models are shown separately for itchy eyes, skin irritation/rash, diarrhea or vomiting, respiratory difficulties, low mood, anxiety/stress, difficulty concentrating, and headache.

| Health Symptoms (Binary Logistic regression) | | | | | |  |
| --- | --- | --- | --- | --- | --- | --- |
| Itchy Eyes |  |  |  |  |  |  |
|  | B | S.E. | Wald | df | Sig. | Exp(B) |
| City (Dhaka) | -0.691 | 0.19 | 13.235 | 1 | <.001 | 0.501 |
| Event (High Air Pollution) | 1.236 | 0.188 | 43.328 | 1 | <.001 | 3.443 |
| Child (Age group) |  |  | 32.973 | 5 | <.001 |  |
| Child (Age group) (25 to 34 years) | 1.846 | 0.747 | 6.117 | 1 | 0.013 | 6.337 |
| Gender |  |  | 10.434 | 2 | 0.005 |  |
| Gender (Male) | -0.431 | 0.181 | 5.68 | 1 | 0.017 | 0.65 |
| Gender (Prefer not to Say) | -1.174 | 0.427 | 7.554 | 1 | 0.006 | 0.309 |
| Monthly Income |  |  | 32.048 | 5 | <.001 |  |
| Monthly Income ($100 to $499) | -0.976 | 0.294 | 11.037 | 1 | <.001 | 0.377 |
| Monthly Income ($500 to $1499) | -1.403 | 0.365 | 14.752 | 1 | <.001 | 0.246 |
| Monthly Income ($1500 to $4000) | -1.323 | 0.514 | 6.621 | 1 | 0.01 | 0.266 |
| Monthly Income (I don’t know) | -0.807 | 0.215 | 14.129 | 1 | <.001 | 0.446 |
| Skin Irritation / Rash |  |  |  |  |  |  |
| City (Dhaka) | -0.666 | 0.192 | 12.08 | 1 | <.001 | 0.514 |
| Event (High Air Pollution) | 0.937 | 0.189 | 24.613 | 1 | <.001 | 2.552 |
| Age Group |  |  | 40.971 | 9 | <.001 |  |
| Child (Age group) |  |  | 43.625 | 5 | <.001 |  |
| Gender |  |  | 16.164 | 2 | <.001 |  |
| Gender (Male) | -0.674 | 0.184 | 13.334 | 1 | <.001 | 0.51 |
| Gender (Prefer not to Say) | -1.121 | 0.431 | 6.773 | 1 | 0.009 | 0.326 |
| Monthly Income |  |  | 39.149 | 5 | <.001 |  |
| Monthly Income ($500 to $1499) | 1.088 | 0.356 | 9.328 | 1 | 0.002 | 2.968 |
| Monthly Income (I don’t know) | -0.787 | 0.216 | 13.215 | 1 | <.001 | 0.455 |
| Diarrhea or Vomiting |  |  |  |  |  |  |
| City (Dhaka) | -0.645 | 0.196 | 10.831 | 1 | <.001 | 0.525 |
| Event (High Air Pollution) | 1.514 | 0.196 | 59.692 | 1 | <.001 | 4.546 |
| Age Group |  |  | 35.872 | 9 | <.001 |  |
| Child (Age group) |  |  | 42.632 | 5 | <.001 |  |
| Child (Age group) (35 to 44 years) | -1.51 | 0.72 | 4.405 | 1 | 0.036 | 0.221 |
| Gender |  |  | 15.965 | 2 | <.001 |  |
| Gender (Male) | -0.696 | 0.187 | 13.91 | 1 | <.001 | 0.499 |
| Gender (Prefer not to Say) | -1.016 | 0.422 | 5.803 | 1 | 0.016 | 0.362 |
| Monthly Income |  |  | 27.692 | 5 | <.001 |  |
| Monthly Income ($500 to $1499) | -1.192 | 0.374 | 10.166 | 1 | 0.001 | 0.304 |
| Monthly Income ($1500 to $4000) | -1.259 | 0.527 | 5.72 | 1 | 0.017 | 0.284 |
| Monthly Income (I don’t know) | -0.996 | 0.221 | 20.262 | 1 | <.001 | 0.369 |
| Respiratory difficulties |  |  |  |  |  |  |
| City (Dhaka) | -0.751 | 0.195 | 14.839 | 1 | <.001 | 0.472 |
| Event (High Air Pollution) | 1.254 | 0.195 | 41.268 | 1 | <.001 | 3.504 |
| Age Group |  |  | 50.967 | 9 | <.001 |  |
| Child (Age group) |  |  | 40.925 | 5 | <.001 |  |
| Gender |  |  | 11.697 | 2 | 0.003 |  |
| Gender (Male) | -0.607 | 0.188 | 10.362 | 1 | 0.001 | 0.545 |
| Gender (Prefer not to Say) | -0.866 | 0.434 | 3.981 | 1 | 0.046 | 0.42 |
| Monthly Income |  |  | 40.447 | 5 | <.001 |  |
| Monthly Income ($500 to $1499) | 1.178 | 0.353 | 11.129 | 1 | <.001 | 3.248 |
| Monthly Income ($1500 to $4000) | 1.357 | 0.504 | 7.242 | 1 | 0.007 | 3.885 |
| Monthly Income (I don’t know) | -0.598 | 0.218 | 7.497 | 1 | 0.006 | 0.55 |
| Low Mood |  |  |  |  |  |  |
| City (Dhaka) | -1.028 | 0.194 | 28.165 | 1 | <.001 | 0.358 |
| Event (High Air Pollution) | 0.944 | 0.196 | 23.255 | 1 | <.001 | 2.569 |
| Age Group |  |  | 32.63 | 9 | <.001 |  |
| Child (Age group) |  |  | 52.734 | 5 | <.001 |  |
| Child (Age group) (25 to 34 years) | 1.734 | 0.735 | 5.563 | 1 | 0.018 | 5.665 |
| Monthly Income |  |  | 29.774 | 5 | <.001 |  |
| Monthly Income ($500 to $1499) | -1.492 | 0.375 | 15.874 | 1 | <.001 | 0.225 |
| Monthly Income ($1500 to $4000) | -1.501 | 0.544 | 7.63 | 1 | 0.006 | 0.223 |
| Anxiety/ Stress |  |  |  |  |  |  |
| City (Dhaka) | -1.266 | 0.198 | 40.87 | 1 | <.001 | 0.282 |
| Event (High Air Pollution) | 0.782 | 0.199 | 15.508 | 1 | <.001 | 2.187 |
| Age Group |  |  | 44.526 | 9 | <.001 |  |
| Child (Age group) |  |  | 53.229 | 5 | <.001 |  |
| Child (Age group) (25 to 34 years) | 2.03 | 0.776 | 6.849 | 1 | 0.009 | 7.613 |
| Monthly Income |  |  | 35.417 | 5 | <.001 |  |
| Monthly Income ($100 to $499) | -0.584 | 0.298 | 3.828 | 1 | 0.05 | 0.558 |
| Monthly Income ($500 to $1499) | -1.769 | 0.382 | 21.385 | 1 | <.001 | 0.171 |
| Monthly Income ($1500 to $4000) | -1.84 | 0.567 | 10.518 | 1 | 0.001 | 0.159 |
| Concentrating at work or school |  |  |  |  |  |  |
| City (Dhaka) | -1.158 | 0.194 | 35.62 | 1 | <.001 | 0.314 |
| Event (High Air Pollution) | 0.802 | 0.199 | 16.28 | 1 | <.001 | 2.231 |
| Age Group |  |  | 43.343 | 9 | <.001 |  |
| Child (Age group) |  |  | 53.921 | 5 | <.001 |  |
| Child (Age group) (25 to 34 years) | 1.534 | 0.727 | 4.454 | 1 | 0.035 | 4.636 |
| Monthly Income |  |  | 10.612 | 5 | 0.06 |  |
| Monthly Income ($500 to $1499) | 0.775 | 0.377 | 4.23 | 1 | 0.04 | 2.171 |
| Headache |  |  |  |  |  |  |
| City (Dhaka) | -0.623 | 0.19 | 10.746 | 1 | 0.001 | 0.536 |
| Event (High Air Pollution) | 0.612 | 0.191 | 10.258 | 1 | 0.001 | 1.845 |
| Age Group |  |  | 36.085 | 9 | <.001 |  |
| Child (Age group) |  |  | 45.165 | 5 | <.001 |  |
| Gender |  |  | 6.728 | 2 | 0.035 |  |
| Gender (Prefer not to Say) | -1.038 | 0.42 | 6.113 | 1 | 0.013 | 0.354 |
| Monthly Income |  |  | 20.126 | 5 | 0.001 |  |
| Monthly Income ($500 to $1499) | -1.073 | 0.353 | 9.251 | 1 | 0.002 | 0.342 |

Reference categories: City = Delhi; Air Quality = Good air quality; Age Group = 55+ years; Child Age Group = Toddler (<1 year); Gender = Female; Monthly Income = < $100; Parent Status = Yes. The table shows only variables with p-values < 0.05.
