# Supplementary material for: Real-time impacts of air pollution on the health, well-being, and daily life of children and young people in Delhi and Dhaka
Source: PLOS Glob Public Health. 2026 Jun 23;6(6):e0005382. doi: 10.1371/journal.pgph.0005382 (PMC13289869; doi:10.1371/journal.pgph.0005382)
Supplement: S6 Table — Adjusted regression models assessing associations between air quality, demographics, and levels of physical activity. (DOCX) [file pgph.0005382.s013.docx]

**S6 Table: Ordinal logistic regression examining associations between air quality events, demographic factors, and daily physical activity duration.**

This table presents results from an ordinal logistic regression model with daily physical activity duration as the dependent variable. The primary predictor is air quality event (Good Air Quality vs. High Air Pollution), with additional factors including age group, child age group, and monthly income. Threshold parameters for physical activity categories, regression coefficients, standard errors, Wald statistics, significance levels, and 95% confidence intervals are reported.

| Physical Activity (Ordinal Logistic regression) | | | | | | | | |
| --- | --- | --- | --- | --- | --- | --- | --- | --- |
|  |  |  |  |  |  |  |  |  |
|  |  | Estimate | Std. Error | Wald | df | Sig. | 95% Confidence Interval | |
|  |  |  |  |  |  |  | Lower Bound | Upper Bound |
| Threshold | [Physical Activity = 0 Minutes] | -1.173 | 0.486 | 5.825 | 1 | 0.016 | -2.126 | -0.22 |
|  | [Physical Activity = 1-15 Minutes] | 0.503 | 0.486 | 1.075 | 1 | 0.3 | -0.448 | 1.455 |
|  | [Physical Activity = 16-30 Minutes] | 1.602 | 0.488 | 10.763 | 1 | 0.001 | 0.645 | 2.559 |
|  | [Physical Activity = 31-45 Minutes] | 1.777 | 0.489 | 13.208 | 1 | <.001 | 0.819 | 2.735 |
|  | [Physical Activity = 46-60 Minutes] | 1.975 | 0.49 | 16.259 | 1 | <.001 | 1.015 | 2.935 |
| Location | [Event=Good Air Quality] | 1.766 | 0.162 | 119.41 | 1 | <.001 | 1.449 | 2.083 |
|  | [Age Group=10 to 14] | -0.826 | 0.319 | 6.722 | 1 | 0.01 | -1.45 | -0.202 |
|  | [Age Group=15 to 19] | -1.054 | 0.324 | 10.595 | 1 | 0.001 | -1.688 | -0.419 |
|  | [Age Group=20 to 24] | -1.3 | 0.348 | 13.991 | 1 | <.001 | -1.982 | -0.619 |
|  | [Age Group=5 to 9] | -0.984 | 0.478 | 4.246 | 1 | 0.039 | -1.92 | -0.048 |
|  | [Child Age Group=Infant (0)] | 1.075 | 0.32 | 11.275 | 1 | <.001 | 0.448 | 1.703 |
|  | [Child Age Group=Pre-Teen (10 to 14)] | 0.595 | 0.265 | 5.017 | 1 | 0.025 | 0.074 | 1.115 |
|  | [Child Age Group=Teen (15 to 17)] | 0.853 | 0.284 | 9.019 | 1 | 0.003 | 0.296 | 1.41 |
|  | [Monthly Income=$1500 to $4000] | -0.69 | 0.277 | 6.203 | 1 | 0.013 | -1.234 | -0.147 |

The table shows only variables with p-values < 0.05. (reference categories are Age Group : 55+ years, child age group : toddler <1 year, and monthly income < $100; Event: High Air Pollution.
