# Supplementary material for: Real-time impacts of air pollution on the health, well-being, and daily life of children and young people in Delhi and Dhaka
Source: PLOS Glob Public Health. 2026 Jun 23;6(6):e0005382. doi: 10.1371/journal.pgph.0005382 (PMC13289869; doi:10.1371/journal.pgph.0005382)
Supplement: S8 Table — Adjusted odds ratios for the likelihood of experiencing daily activity disruptions during high-pollution periods. (DOCX) [file pgph.0005382.s015.docx]

**S8 Table: Binary logistic regression examining associations between high air pollution, demographic factors, and daily activity disruptions.**

This table presents adjusted odds ratios from separate binary logistic regression models for eight types of daily activity disruptions: being late for school or work, missing school or work, missing meetings or interviews, missing healthcare appointments, missing social meetings with friends or family, lacking enough food, lacking access to clean drinking water, and needing more family assistance. The primary predictor is air quality event (Good Air Quality vs. High Air Pollution), with demographic variables including city, age group, child age group, gender, and monthly income. Coefficients, standard errors, Wald statistics, significance levels, and odds ratios (Exp[B]) are reported for variables with p < 0.05.

| Daily Activity Disruptions (Binary Logistic regression) | | | | | | |
| --- | --- | --- | --- | --- | --- | --- |
|  | B | S.E. | Wald | df | Sig. | Exp(B) |
| Late for school or work |  |  |  |  |  |  |
| City (Dhaka) | -0.576 | 0.194 | 8.851 | 1 | 0.003 | 0.562 |
| Event (High Air Pollution) | 1.527 | 0.194 | 62.089 | 1 | <.001 | 4.603 |
| Age Group |  |  | 17.968 | 9 | 0.036 |  |
| Child (Age group) |  |  | 52.824 | 5 | <.001 |  |
| Child (Age group) (20 to 24 years) | 1.82 | 0.794 | 5.247 | 1 | 0.022 | 6.17 |
| Child (Age group) (25 to 34 years) | 2.434 | 0.807 | 9.097 | 1 | 0.003 | 11.403 |
| Gender |  |  | 14.62 | 2 | <.001 |  |
| Gender (Male) | -0.564 | 0.187 | 9.126 | 1 | 0.003 | 0.569 |
| Gender (Prefer not to Say) | -1.38 | 0.451 | 9.376 | 1 | 0.002 | 0.252 |
| Monthly Income |  |  | 22.928 | 5 | <.001 |  |
| Monthly Income ($100 to $499) | -0.77 | 0.302 | 6.51 | 1 | 0.011 | 0.463 |
| Monthly Income ($500 to $1499) | -0.728 | 0.358 | 4.135 | 1 | 0.042 | 0.483 |
| Monthly Income ($1500 to $4000) | -1.563 | 0.544 | 8.267 | 1 | 0.004 | 0.209 |
| Monthly Income (I don’t know) | -0.574 | 0.219 | 6.857 | 1 | 0.009 | 0.563 |
| Missed school of work |  |  |  |  |  |  |
| City (Dhaka) | -1.029 | 0.198 | 26.885 | 1 | <.001 | 0.357 |
| Event (High Air Pollution) | 1.323 | 0.194 | 46.484 | 1 | <.001 | 3.756 |
| Age Group |  |  | 24.683 | 9 | 0.003 |  |
| Child (Age group) |  |  | 48.261 | 5 | <.001 |  |
| Child (Age group) (25 to 34 years) | 1.571 | 0.755 | 4.331 | 1 | 0.037 | 4.812 |
| Gender |  |  | 8.064 | 2 | 0.018 |  |
| Gender (Prefer not to Say) | -1.272 | 0.463 | 7.539 | 1 | 0.006 | 0.28 |
| Monthly Income |  |  | 45.784 | 5 | <.001 |  |
| Monthly Income ($500 to $1499) | 0.76 | 0.357 | 4.518 | 1 | 0.034 | 2.137 |
| Monthly Income ($1500 to $4000) | -2.509 | 0.622 | 16.26 | 1 | <.001 | 0.081 |
| Monthly Income (I don’t know) | -0.883 | 0.221 | 15.915 | 1 | <.001 | 0.414 |
| Missed meeting or interview |  |  |  |  |  |  |
| City (Dhaka) | -0.916 | 0.196 | 21.764 | 1 | <.001 | 0.4 |
| Event (High Air Pollution) | 1.47 | 0.197 | 55.922 | 1 | <.001 | 4.348 |
| Age Group |  |  | 33.843 | 9 | <.001 |  |
| Child (Age group) |  |  | 45.787 | 5 | <.001 |  |
| Child (Age group) (35 to 44 years) | -1.641 | 0.714 | 5.275 | 1 | 0.022 | 0.194 |
| Gender |  |  | 10.112 | 2 | 0.006 |  |
| Gender (Prefer not to Say) | -1.469 | 0.482 | 9.286 | 1 | 0.002 | 0.23 |
| Monthly Income |  |  | 33.804 | 5 | <.001 |  |
| Monthly Income ($500 to $1499) | 1.245 | 0.361 | 11.925 | 1 | <.001 | 3.474 |
| Monthly Income ($1500 to $4000) | -1.446 | 0.55 | 6.898 | 1 | 0.009 | 0.236 |
| Monthly Income (I don’t know) | -0.495 | 0.218 | 5.18 | 1 | 0.023 | 0.609 |
| Missed Healthcare appointment |  |  |  |  |  |  |
| City (Dhaka) | -0.842 | 0.196 | 18.531 | 1 | <.001 | 0.431 |
| Event (High Air Pollution) | 1.49 | 0.198 | 56.891 | 1 | <.001 | 4.437 |
| Age Group |  |  | 27.676 | 9 | 0.001 |  |
| Child (Age group) |  |  | 42.455 | 5 | <.001 |  |
| Child (Age group) (35 to 44 years) | -1.576 | 0.712 | 4.897 | 1 | 0.027 | 0.207 |
| Gender |  |  | 7.955 | 2 | 0.019 |  |
| Gender (Prefer not to Say) | -1.227 | 0.478 | 6.583 | 1 | 0.01 | 0.293 |
| Monthly Income |  |  | 16.504 | 5 | 0.006 |  |
| Monthly Income ($500 to $1499) | -0.791 | 0.373 | 4.511 | 1 | 0.034 | 0.453 |
| Monthly Income ($1500 to $4000) | -2.028 | 0.612 | 10.994 | 1 | <.001 | 0.132 |
| Missed meeting up with friends / family | | | | | | |
| City (Dhaka) | -0.603 | 0.188 | 10.254 | 1 | 0.001 | 0.547 |
| Event (High Air Pollution) | 1.373 | 0.19 | 52.492 | 1 | <.001 | 3.948 |
| Age Group |  |  | 24.146 | 9 | 0.004 |  |
| Child (Age group) |  |  | 36.561 | 5 | <.001 |  |
| Child (Age group) (25 to 34 years) | 1.92 | 0.772 | 6.183 | 1 | 0.013 | 6.819 |
| Gender |  |  | 11.626 | 2 | 0.003 |  |
| Gender (Male) | -0.529 | 0.181 | 8.54 | 1 | 0.003 | 0.589 |
| Gender (Prefer not to Say) | -1.054 | 0.422 | 6.223 | 1 | 0.013 | 0.349 |
| Monthly Income ($100 to $499) | -0.581 | 0.293 | 3.933 | 1 | 0.047 | 0.559 |
| Monthly Income ($500 to $1499) | -0.851 | 0.362 | 5.539 | 1 | 0.019 | 0.427 |
| Monthly Income ($1500 to $4000) | -1.045 | 0.513 | 4.142 | 1 | 0.042 | 0.352 |
| Not have enough food |  |  |  |  |  |  |
| City (Dhaka) | -0.75 | 0.193 | 15.119 | 1 | <.001 | 0.472 |
| Event (High Air Pollution) | 0.629 | 0.196 | 10.3 | 1 | 0.001 | 1.876 |
| Age Group |  |  | 51.559 | 9 | <.001 |  |
| Child (Age group) |  |  | 36.829 | 5 | <.001 |  |
| Gender |  |  | 10.679 | 2 | 0.005 |  |
| Gender (Prefer not to Say) | -1.513 | 0.472 | 10.281 | 1 | 0.001 | 0.22 |
| Monthly Income |  |  | 16.324 | 5 | 0.006 |  |
| Monthly Income ($1500 to $4000) | -2.015 | 0.636 | 10.019 | 1 | 0.002 | 0.133 |
| No access clean drinking water |  |  |  |  |  |  |
| City (Dhaka) | -0.792 | 0.198 | 15.989 | 1 | <.001 | 0.453 |
| Event (High Air Pollution) | 1.569 | 0.198 | 62.982 | 1 | <.001 | 4.802 |
| Age Group |  |  | 32.459 | 9 | <.001 |  |
| Child (Age group) |  |  | 36.326 | 5 | <.001 |  |
| Gender |  |  | 15.512 | 2 | <.001 |  |
| Gender (Male) | -0.433 | 0.189 | 5.242 | 1 | 0.022 | 0.649 |
| Gender (Prefer not to Say) | -1.786 | 0.484 | 13.605 | 1 | <.001 | 0.168 |
| Monthly Income |  |  | 35.934 | 5 | <.001 |  |
| Monthly Income ($500 to $1499) | 1.366 | 0.37 | 13.607 | 1 | <.001 | 3.921 |
| Monthly Income (I don’t know) | -0.464 | 0.218 | 4.502 | 1 | 0.034 | 0.629 |
| Need for more family assistance |  |  |  |  |  |  |
| City (Dhaka) | -0.76 | 0.196 | 15.101 | 1 | <.001 | 0.468 |
| Event (High Air Pollution) | 1.505 | 0.195 | 59.59 | 1 | <.001 | 4.502 |
| Age Group |  |  | 33.644 | 9 | <.001 |  |
| Child (Age group) |  |  | 30.331 | 5 | <.001 |  |
| Child (Age group) (25 to 34 years) | 1.741 | 0.781 | 4.975 | 1 | 0.026 | 5.704 |
| Gender |  |  | 11.988 | 2 | 0.002 |  |
| Gender (Male) | -0.504 | 0.188 | 7.179 | 1 | 0.007 | 0.604 |
| Gender (Prefer not to Say) | -1.242 | 0.435 | 8.136 | 1 | 0.004 | 0.289 |
| Monthly Income |  |  | 35.521 | 5 | <.001 |  |
| Monthly Income ($500 to $1499) | 0.954 | 0.356 | 7.181 | 1 | 0.007 | 2.595 |
| Monthly Income ($1500 to $4000) | -1.932 | 0.577 | 11.207 | 1 | <.001 | 0.145 |
| Monthly Income (I don’t know) | -0.551 | 0.217 | 6.456 | 1 | 0.011 | 0.576 |

Reference categories: Event = Good Air Quality, City= Delhi, age group = 55+ years, child age group = toddler <1 year, gender =female, and monthly income < $100. Table shows only the variables that were statistically significant.
