# Supplementary material for: Real-time impacts of air pollution on the health, well-being, and daily life of children and young people in Delhi and Dhaka
Source: PLOS Glob Public Health. 2026 Jun 23;6(6):e0005382. doi: 10.1371/journal.pgph.0005382 (PMC13289869; doi:10.1371/journal.pgph.0005382)
Supplement: Attachment — Submitted filename: Response to Reviewers.docx [file pgph.0005382.s022.docx]

**Response to Reviewers**

Manuscript ID: PGPH-D-25-03088
Title: *Real-time impacts of air pollution on the health, well-being, and daily life of children and young people in Delhi and Dhaka*

Dear Dr. Pant and Reviewers,

We thank you for your careful and constructive review. We have revised the manuscript substantially in response to your comments. All changes are reflected in the tracked version. Below we provide point-by-point responses.

**Editor Comment**

| **Comment** | **Response** |
| --- | --- |
| Hindi survey appears to refer to heatwaves rather than air pollution. | Thank you for identifying this. An incorrect supplementary file (from a previous heatwave study) was inadvertently uploaded. The dataset analyzed corresponds to the correct air pollution survey particularly . We have replaced the incorrect Hindi file with the correct instrument and verified all supplementary materials (S2_Text). Screen 11 now says: **“क्या उच्च वायु प्रदूषण के कारण…”** Screen 12 now says: **“उच्च वायु प्रदूषण के बारे में आप कितने चिंतित हैं?”** and Screen 13 now says: **“उच्च वायु प्रदूषण के लिए आपके समुदाय की तैयारी…”.** We sincerely apologize for the error. |

**Reviewer 1**

| **Comment** | **Response** |
| --- | --- |
| Conceptual framework underdeveloped. | Thank you for this comment. We have added a paragraph in the **Discussion section (p. 38–39, Lines 705–716)** that outlines a multi-level conceptual framework linking PM₂.₅ exposure to biological mechanisms, psychological effects, behavioural responses, and broader structural factors. This addition clarifies the pathways through which air pollution may affect health and daily activities and strengthens the interpretation of our findings. |
| Self-selection bias from digital recruitment. | We expanded the Limitations to discuss digital access bias, underrepresentation of low-income CYP, and potential self-selection based on pollution awareness (Page 41, Lines 750-758). |
| No a priori power calculation. | We have clarified in the Methods section that the event-triggered design did not allow for an a priori power calculation, as recruitment was determined by real-time air quality thresholds (Methods, p. 12, lines 204-210). We also note that adjusted odds ratios and full regression estimates are reported to allow assessment of effect size and precision (p. 12, lines 206–210; Supplementary Tables S1-S9). |
| Discussion repeats numbers rather than interpreting findings. | We have revised the Discussion to focus less on repeating numerical results and to provide clearer interpretation of the differences observed between Delhi and Dhaka. In the revised text, we discuss how variations in pollution sources, exposure conditions, urban infrastructure, and service systems may help explain why some symptoms and daily disruptions differed between the two cities. We also highlight the role of behavioural responses, such as reduced outdoor activity or delayed travel, in shaping how pollution episodes affect daily life. These revisions aim to strengthen the analytical interpretation of the findings rather than simply restating the results (P.31-39, Lines 560-716) |
| Why food insecurity in Dhaka but not Delhi? | We have revised the Discussion to clarify this difference. The text now suggests that the reported food insecurity in Dhaka may reflect underlying structural and socioeconomic vulnerabilities, whereas comparatively stronger infrastructure and service systems in Delhi may buffer some of these disruptions (P. 36, Lines 654-663). |
| Limited engagement with policy frameworks. | We have revised the Public Perceptions and Policy Implications section to better connect our findings with existing air quality policy frameworks. The revised text now refers to international guidance (e.g., WHO recommendations) and regional policy efforts in South Asia, and explains how participants’ suggested solutions align with these policy priorities (P.39-40, Lines 720-731). |
| Seasonal confounding | Thank you for this comment. We have added a discussion of potential seasonal confounders (e.g., temperature, pollen, viral illness, and temperature inversions) in the Limitations section and clarified that these factors may partly influence the observed differences between periods. (P.42, Lines 768-776). |

**Reviewer #2**

| **Comment** | **Response** |
| --- | --- |
| Hindi survey mismatch (heatwave vs air pollution). | Confirmed upload error. Correct air pollution survey now uploaded. Dataset analyzed corresponds to correct instrument (S2_Text). |
| Limited literature review. | We have revised the Background section to strengthen the review of relevant literature and clarify how this study builds on existing research on the health and well-being impacts of air pollution, particularly among children and young people (P.4-6, Lines 75-77, Lines 91-94). |
| Generic response categories (“good”, “ok”). | Thank you for this suggestion. The survey was designed as a rapid-response digital questionnaire intended to minimize respondent burden during short data collection windows. For this reason, brief categorical measures of general feelings and sleep quality were used. We acknowledge that more granular scales could provide additional detail and have added this point to the Limitations section as a consideration for future research (P.40, Lines 739-744). |
| Additional seasonal covariates needed. | Thank you for this comment. We have added a discussion of potential seasonal confounders (e.g., temperature, pollen, viral illness, and temperature inversions) in the Limitations section and clarified that these factors may partly influence the observed differences between periods (P.42, Lines 768-776). |
| Explain age, income, and gender variation. | We have expanded the Discussion section to provide possible explanations for the observed differences by age (P.33, Lines 602-618), income (P.34, Lines 619-628), and gender (P.34-35, Lines 631-636). The revised text now includes brief interpretations of these patterns. |
| Why greater health burden in Delhi? | We have expanded the Discussion section to include possible explanations for the higher reported health burden in Delhi, including differences in pollution sources, atmospheric conditions, and exposure intensity between the two cities (P.36-37, Lines 654-678). |
| Younger parents reporting greater mental impacts. | We have expanded the Discussion section to include possible explanations for this finding. The revised text now discusses greater awareness of child mental health among younger parents and the potential vulnerability of younger children to pollution-related stressors (P. 33-34, Lines 610-618). |

**Minor Comments**

1. Line 56: population breathes air with pollutant concentrations that exceed the recommended health-based limits
2. Line 111: add closing bracket after “m3”
3. Line 261: What is an “in vivo strategy” in this case?
4. Line 467: add “reported during good air quality”
5. Line 467 – 468: The order of reduction is from good air quality to bad air quality, which is not intuitive from the way the sentence is written.

**Minor Comment responses:**

All minor corrections (wording, bracket closure, clarification of “in vivo strategy i.e., (i.e., using participants’ own words or phrases as codes),” sentence structure revisions) have been addressed (P.3, Line 56; P.6, Line 114; P.15, Lines 271-272; P.26, Line 478; P.26, Lines 478-479).

**Closing Statement**

We are grateful for the reviewers’ thoughtful feedback. The manuscript has been strengthened through:

- Clearer conceptual framing
- Expanded policy relevance
- Deeper interpretation of findings
- Improved limitations discussion
- Correction of supplementary materials

We hope the revised manuscript now meets the journal’s publication criteria and appreciate your consideration.

Sincerely,
Constance Bwire
On behalf of all co-authors
