# Supplementary material for: Real-time impacts of air pollution on the health, well-being, and daily life of children and young people in Delhi and Dhaka
Source: PLOS Glob Public Health. 2026 Jun 23;6(6):e0005382. doi: 10.1371/journal.pgph.0005382 (PMC13289869; doi:10.1371/journal.pgph.0005382)
Supplement: Attachment — Submitted filename: PGPH-D-25-03088.docx [file pgph.0005382.s020.docx]

This cross-sectional study investigates the real-time impacts of air pollution (PM₂.₅) on the health, well-being, and daily activities of children and young people (CYP) aged 13–29 years, as well as parents reporting for children under 18, in Delhi, India, and Dhaka, Bangladesh. Using a digital survey deployed during verified high-pollution periods (PM₂.₅ > 125.5 µg/m³) in January 2025 and good air quality periods (PM₂.₅ ≤ 35.4 µg/m³) in March 2025, the authors collected 814 responses (Delhi: 365; Dhaka: 449). Quantitative analyses (chi-square tests and regression models adjusted for demographics) show associations between high pollution and increased reports of symptoms like itchy eyes, respiratory difficulties, headaches, low mood, and anxiety, as well as disruptions such as reduced physical activity and missing school/work. Qualitative responses from open-ended questions were thematically analyzed, revealing CYP suggestions clustered around cleaner environments, community values, health/education improvements, pollution/technology solutions, and other ideas. The study emphasizes CYP-centered evidence for equitable air quality management in urban South Asia. However, while the descriptive and comparative findings are interesting, the paper could be strengthened through deeper analytical interpretation, improved methodological rigor, and clearer contextualization of limitations.

1. The conceptual framework connecting PM₂.₅ exposure with the measured outcomes (health, well-being, daily activities) is underdeveloped. The authors should integrate a mechanistic or behavioral model to strengthen interpretability.
2. The recruitment via paid digital advertisements introduces strong self-selection bias. The authors acknowledge this but should more clearly discuss how this affects representativeness, especially for low-income children and youth.
3. There is mention of data collection “without a priori power calculation.” This weakens inferential claims. Consider including post-hoc power or effect size estimation to contextualize statistical significance.
4. While differences between pollution periods are statistically significant, many results are descriptive. The discussion often repeats numbers rather than interpreting why particular symptoms or disruptions differ between cities.
5. Why did Dhaka show food insecurity effects while Delhi did not?
6. The discussion does not sufficiently engage with existing regional or national air quality management frameworks. Linking findings to current policy debates would enhance the manuscript’s relevance.
7. Data collection occurred in winter (January: high pollution, cooler weather) vs. spring (March: good air, warmer). This introduces potential confounders like temperature inversions, pollen/allergens, or seasonal illnesses, which could explain differences in symptoms rather than PM₂.₅ alone. Regression adjusts for demographics but not seasonal/environmental variables. Discuss and justify why these were not controlled for.

The study offers valuable, timely insights into air pollution's real-time effects on CYP, with strong potential for impact in urban health policy. However, methodological limitations need addressing to strengthen validity. Revise to include more robust controls, clearer limitations, and suggestions for future work.
