# Supplementary material for: Real-time impacts of air pollution on the health, well-being, and daily life of children and young people in Delhi and Dhaka
Source: PLOS Glob Public Health. 2026 Jun 23;6(6):e0005382. doi: 10.1371/journal.pgph.0005382 (PMC13289869; doi:10.1371/journal.pgph.0005382)
Supplement: Peer review history

# Peer review: Decision Letter 0

**DOI:** 10.1371/journal.pgph.0005382.r001

**Authors:** Pallavi Pant

15 Dec 2025

PGPH-D-25-03088

Real-time impacts of air pollution on the health, well-being, and daily life of children and young people in Delhi and Dhaka

PLOS Global Public Health

Dear Dr. Bwire,

Thank you for submitting your manuscript to PLOS Global Public Health. After careful consideration, we feel that it has merit but does yet not fully meet PLOS Global Public Health’s publication criteria as it currently stands. Therefore, we invite you to submit a revised version of the manuscript that addresses the points raised during the review process.

This is an interesting study design in cities with high levels of air pollution. As such there is likely to be broad interest in the findings. We request the authors to respond to the detailed queries raised by the reviewers. In case of any queries, please feel free to reach out to us.

In particular, note that the Hindi survey attached in the paper is asking about the impact of heatwaves on disruption of activity, not about the impact of air pollution. Similarly, questions about community preparedness are pertaining to heat waves, not air pollution. Was there perhaps a mix up in uploading the survey files?

Please submit your revised manuscript by Jan 29 2026 11:59PM. If you will need more time than this to complete your revisions, please reply to this message or contact the journal office at globalpubhealth@plos.org. When you're ready to submit your revision, log on to https://www.editorialmanager.com/pgph/ and select the 'Submissions Needing Revision' folder to locate your manuscript file.

Please include the following items when submitting your revised manuscript:

- A letter that responds to each point raised by the editor and reviewer(s). You should upload this letter as a separate file labeled 'Response to Reviewers'.
- A marked-up copy of your manuscript that highlights changes made to the original version. You should upload this as a separate file labeled 'Revised Manuscript with Track Changes'.
- An unmarked version of your revised paper without tracked changes. You should upload this as a separate file labeled 'Manuscript'.

Guidelines for resubmitting your figure files are available below the reviewer comments at the end of this letter.

We look forward to receiving your revised manuscript.

Kind regards,

Pallavi Pant, PhD

Academic Editor

PLOS Global Public Health

Journal Requirements:

If the reviewer comments include a recommendation to cite specific previously published works, please review and evaluate these publications to determine whether they are relevant and should be cited. There is no requirement to cite these works unless the editor has indicated otherwise.

Additional Editor Comments (if provided):

[Note: HTML markup is below. Please do not edit.]

Reviewers' comments:

Reviewer's Responses to Questions

**Comments to the Author**

1. Does this manuscript meet PLOS Global Public Health’s publication criteria? Is the manuscript technically sound, and do the data support the conclusions? The manuscript must describe methodologically and ethically rigorous research with conclusions that are appropriately drawn based on the data presented.

Reviewer #1: Partly

Reviewer #2: Partly

**********

2. Has the statistical analysis been performed appropriately and rigorously?

Reviewer #1: No

Reviewer #2: Yes

**********

3. Have the authors made all data underlying the findings in their manuscript fully available (please refer to the Data Availability Statement at the start of the manuscript PDF file)?

The PLOS Data policy requires authors to make all data underlying the findings described in their manuscript fully available without restriction, with rare exception. The data should be provided as part of the manuscript or its supporting information, or deposited to a public repository. For example, in addition to summary statistics, the data points behind means, medians and variance measures should be available. If there are restrictions on publicly sharing data—e.g. participant privacy or use of data from a third party—those must be specified.

Reviewer #1: Yes

Reviewer #2: Yes

**********

4. Is the manuscript presented in an intelligible fashion and written in standard English?

PLOS Global Public Health does not copyedit accepted manuscripts, so the language in submitted articles must be clear, correct, and unambiguous. Any typographical or grammatical errors should be corrected at revision, so please note any specific errors here.

Reviewer #1: Yes

Reviewer #2: Yes

**********

5. Review Comments to the Author

Please use the space provided to explain your answers to the questions above. You may also include additional comments for the author, including concerns about dual publication, research ethics, or publication ethics. (Please upload your review as an attachment if it exceeds 20,000 characters)

Reviewer #1: This cross-sectional study investigates the real-time impacts of air pollution (PM₂.₅) on the health, well-being, and daily activities of children and young people (CYP) aged 13–29 years, as well as parents reporting for children under 18, in Delhi, India, and Dhaka, Bangladesh. Using a digital survey deployed during verified high-pollution periods (PM₂.₅ > 125.5 µg/m³) in January 2025 and good air quality periods (PM₂.₅ ≤ 35.4 µg/m³) in March 2025, the authors collected 814 responses (Delhi: 365; Dhaka: 449). Quantitative analyses (chi-square tests and regression models adjusted for demographics) show associations between high pollution and increased reports of symptoms like itchy eyes, respiratory difficulties, headaches, low mood, and anxiety, as well as disruptions such as reduced physical activity and missing school/work. Qualitative responses from open-ended questions were thematically analyzed, revealing CYP suggestions clustered around cleaner environments, community values, health/education improvements, pollution/technology solutions, and other ideas. The study emphasizes CYP-centered evidence for equitable air quality management in urban South Asia. However, while the descriptive and comparative findings are interesting, the paper could be strengthened through deeper analytical interpretation, improved methodological rigor, and clearer contextualization of limitations.

1. The conceptual framework connecting PM₂.₅ exposure with the measured outcomes (health, well-being, daily activities) is underdeveloped. The authors should integrate a mechanistic or behavioral model to strengthen interpretability.

2. The recruitment via paid digital advertisements introduces strong self-selection bias. The authors acknowledge this but should more clearly discuss how this affects representativeness, especially for low-income children and youth.

3. There is mention of data collection “without a priori power calculation.” This weakens inferential claims. Consider including post-hoc power or effect size estimation to contextualize statistical significance.

4. While differences between pollution periods are statistically significant, many results are descriptive. The discussion often repeats numbers rather than interpreting why particular symptoms or disruptions differ between cities.

5. Why did Dhaka show food insecurity effects while Delhi did not?

6. The discussion does not sufficiently engage with existing regional or national air quality management frameworks. Linking findings to current policy debates would enhance the manuscript’s relevance.

7. Data collection occurred in winter (January: high pollution, cooler weather) vs. spring (March: good air, warmer). This introduces potential confounders like temperature inversions, pollen/allergens, or seasonal illnesses, which could explain differences in symptoms rather than PM₂.₅ alone. Regression adjusts for demographics but not seasonal/environmental variables. Discuss and justify why these were not controlled for.

The study offers valuable, timely insights into air pollution's real-time effects on CYP, with strong potential for impact in urban health policy. However, methodological limitations need addressing to strengthen validity. Revise to include more robust controls, clearer limitations, and suggestions for future work.

Reviewer #2: Major comments –

1. The Hindi survey attached in the paper is asking about the impact of heatwaves on disruption of activity, not about the impact of air pollution. Similarly, questions about community preparedness are pertaining to heat waves, not air pollution. It is possible that the wrong survey file was attached along with the manuscript. In that case, this error should be rectified. In case the Hindi survey uploaded is the one used for data collection for this paper, then this analysis is incorrect and the paper should not be published.

2. While there is a comparison of the results to the literature in the Discussion section, there is no review of literature related to the paper.

3. Response categories like “good” and “ok” are very generic responses to how one is feeling or about sleep quality. In order to get a more granular estimate, perhaps one alternative could be to ask questions ranking duration and quality of sleep on a scale of 1 to 10.

4. Additional covariates that determine well-being and quality of sleep should be taken into account in the regression. For example, in Delhi, December is in the middle of the winter season while March marks the onset of spring in the city. For a person who dislikes the winter, these seasonal factors could affect their outcome variables. Adding more predictors will help to ascertain with greater accuracy the role that air pollution plays in affecting these outcomes.

5. Line 368 – 367: Potential reasons/ hypotheses explaining why sleep quality of infants and pre-teens was affected. Similar arguments should be provided for income groups and gender variations in further detail in the discussion section.

6. Line 420 – 422: Potential reasons/ hypotheses explaining why the health burden in Delhi is greater than in Dhaka should be provided in further detail in the discussion section. Existing literature may offer some insight.

7. Line 445 – 448: Potential reasons/ hypotheses explaining why younger parents perceive greater emotional and mental impacts on their children in further detail in the discussion section. Is it because the parents are more sensitive to the mental health of their children or are their children more susceptible to air pollution?

Minor comments –

1. Line 56: population breathes air with pollutant concentrations that exceed the recommended health-based limits

2. Line 111: add closing bracket after “m3”

3. Line 261: What is an “in vivo strategy” in this case?

4. Line 467: add “reported during good air quality”

5. Line 467 – 468: The order of reduction is from good air quality to bad air quality, which is not intuitive from the way the sentence is written.

**********

6. PLOS authors have the option to publish the peer review history of their article (what does this mean?). If published, this will include your full peer review and any attached files.

**Do you want your identity to be public for this peer review?**   If you choose “no”, your identity will remain anonymous but your review may still be made public.

For information about this choice, including consent withdrawal, please see our Privacy Policy.

Reviewer #1: No

Reviewer #2: **Yes:**   Annanya Mahajan

**********

[NOTE: If reviewer comments were submitted as an attachment file, they will be attached to this email and accessible via the submission site. Please log into your account, locate the manuscript record, and check for the action link "View Attachments". If this link does not appear, there are no attachment files.]

-->Figure Resubmissions:

While revising your submission, we strongly recommend that you use PLOS’s NAAS tool (https://ngplosjournals.pagemajik.ai/artanalysis) to test your figure files. NAAS can convert your figure files to the TIFF file type and meet basic requirements (such as print size, resolution), or provide you with a report on issues that do not meet our requirements and that NAAS cannot fix.

After uploading your figures to PLOS’s NAAS tool - https://ngplosjournals.pagemajik.ai/artanalysis, NAAS will process the files provided and display the results in the "Uploaded Files" section of the page as the processing is complete. If the uploaded figures meet our requirements (or NAAS is able to fix the files to meet our requirements), the figure will be marked as "fixed" above. If NAAS is unable to fix the files, a red "failed" label will appear above. When NAAS has confirmed that the figure files meet our requirements, please download the file via the download option, and include these NAAS processed figure files when submitting your revised manuscript.

Attachment Submitted filename: PGPH-D-25-03088.docx

Attachment Submitted filename: PGPH-D-25-03088.docx

# Author comment: Author response to Decision Letter 1

**DOI:** 10.1371/journal.pgph.0005382.r002

7 Mar 2026

Attachment Submitted filename: Response to Reviewers.docx

# Peer review: Decision Letter 1

**DOI:** 10.1371/journal.pgph.0005382.r003

**Authors:** Pallavi Pant, Julia Robinson

24 May 2026

Real-time impacts of air pollution on the health, well-being, and daily life of children and young people in Delhi and Dhaka

PGPH-D-25-03088R1

Dear Dr. Bwire,

We are pleased to inform you that your manuscript 'Real-time impacts of air pollution on the health, well-being, and daily life of children and young people in Delhi and Dhaka' has been provisionally accepted for publication in PLOS Global Public Health.

Before your manuscript can be formally accepted you will need to complete some formatting changes, which you will receive in a follow up email. A member of our team will be in touch with a set of requests.

Please note that your manuscript will not be scheduled for publication until you have made the required changes, so a swift response is appreciated.

IMPORTANT: The editorial review process is now complete. PLOS will only permit corrections to spelling, formatting or significant scientific errors from this point onwards. Requests for major changes, or any which affect the scientific understanding of your work, will cause delays to the publication date of your manuscript.

If your institution or institutions have a press office, please notify them about your upcoming paper to help maximize its impact. If they'll be preparing press materials, please inform our press team as soon as possible -- no later than 48 hours after receiving the formal acceptance. Your manuscript will remain under strict press embargo until 2 pm Eastern Time on the date of publication. For more information, please contact globalpubhealth@plos.org.

Thank you again for supporting Open Access publishing; we are looking forward to publishing your work in PLOS Global Public Health.

Best regards,

Julia Robinson

Executive Editor

PLOS Global Public Health

***********************************************************

Reviewer Comments (if any, and for reference):

Reviewer's Responses to Questions

**Comments to the Author**

1. If the authors have adequately addressed your comments raised in a previous round of review and you feel that this manuscript is now acceptable for publication, you may indicate that here to bypass the “Comments to the Author” section, enter your conflict of interest statement in the “Confidential to Editor” section, and submit your "Accept" recommendation.

Reviewer #2: All comments have been addressed

**********

2. Does this manuscript meet PLOS Global Public Health’s publication criteria? Is the manuscript technically sound, and do the data support the conclusions? The manuscript must describe methodologically and ethically rigorous research with conclusions that are appropriately drawn based on the data presented.

Reviewer #2: Yes

**********

3. Has the statistical analysis been performed appropriately and rigorously?

Reviewer #2: Yes

**********

4. Have the authors made all data underlying the findings in their manuscript fully available (please refer to the Data Availability Statement at the start of the manuscript PDF file)?

The PLOS Data policy requires authors to make all data underlying the findings described in their manuscript fully available without restriction, with rare exception. The data should be provided as part of the manuscript or its supporting information, or deposited to a public repository. For example, in addition to summary statistics, the data points behind means, medians and variance measures should be available. If there are restrictions on publicly sharing data—e.g. participant privacy or use of data from a third party—those must be specified.

Reviewer #2: Yes

**********

5. Is the manuscript presented in an intelligible fashion and written in standard English?

PLOS Global Public Health does not copyedit accepted manuscripts, so the language in submitted articles must be clear, correct, and unambiguous. Any typographical or grammatical errors should be corrected at revision, so please note any specific errors here.

Reviewer #2: Yes

**********

6. Review Comments to the Author

Please use the space provided to explain your answers to the questions above. You may also include additional comments for the author, including concerns about dual publication, research ethics, or publication ethics. (Please upload your review as an attachment if it exceeds 20,000 characters)

Reviewer #2: All my comments from the previous round of review that could have been addressed, have been addressed. The manuscript is now suitable for publication.

**********

7. PLOS authors have the option to publish the peer review history of their article (what does this mean?). If published, this will include your full peer review and any attached files.

**Do you want your identity to be public for this peer review?**   If you choose “no”, your identity will remain anonymous but your review may still be made public.

For information about this choice, including consent withdrawal, please see our Privacy Policy.

Reviewer #2: No

**********
